# Advances in the pathophysiology and treatment of anaemia in multiple myeloma

**DOI:** 10.1097/MOH.0000000000000911

**Published:** 2026-02-11

**Authors:** Nicola Giuliani, Benedetta dalla Palma, Laura Notarfranchi

**Affiliations:** a Department of Medicine and Surgery, University of Parma, Multiple Myeloma Research Unit, Hematological Hospital Parma; b Hematology Unit Hematological Hospital Parma, Parma; c UOC Ematologia Aziendale, Azienda USL Toscana Nordovest, Ospedale Nuovo Apuane, Massa Carrara, Italy

**Keywords:** anaemia, erythropoietin, immunotherapy, multiple myeloma, sotatercept

## Abstract

**Purpose of review:**

Anaemia is a common sign in multiple myeloma due to multifactorial mechanisms. Apart from the well known factors related to the disease, more recently, the use of several new drugs as the immunotherapeutic ones indicates the emerging role of drug-related mechanisms in the pathophysiology of anaemia in multiple myeloma patients.

**Recent findings:**

Anaemia associated with immunomodulatory drugs may result from both direct effect on hematopoietic progenitor cells and indirect one mediated by cytokine modulation within the bone marrow microenvironment. The CD38 expression by erythroid lineage cells, suggests that anti-CD38 antibodies may induce apoptosis or functional inhibition of these progenitors, leading to reductions in erythropoiesis. Recent clinical trials also reported ad high incidence of anaemia in multiple myeloma patients treated either with bispecific antibodies or CAR-T cells.

Treatment of anaemia in multiple myeloma patients included the use of red blood cell transfusion and erythropoietin-stimulating agents but recently novel agents were under investigation as the activin receptor fusion proteins.

**Summary:**

The use of the new immunotherapeutic drugs in multiple myeloma patients is associated with an increased incidence of anaemia that should be considered by clinicians particularly in the management of those patients in which coexist other anaemia-related factors.

## INTRODUCTION

One of the main common features of multiple myeloma (MM) is anaemia characterized by signs and symptoms as fatigue, weakness, shortness of breath and pallor [1]. Different mechanisms are involved in the pathogenesis of anaemia in MM patients including increasing apoptosis of erythroid progenitors due to the secretion of TNF and related cytokines (i.e. TRAIL) [2]. Bone marrow replacement by plasma cells, a relative erythropoietin deficiency for renal insufficiency, but also bleeding. Recently the possible role of the chemokine CCL3 has been also underlined showing that elevated CCL3 in the bone marrow plasma of myeloma further inhibited the erythropoiesis of haemopoietic stem cells (HSC)s via activation of CCL3/CCR1/p38 signalling and suppressed GATA1 expression in HSCs and erythroid progenitors [3].

In addition, many MM patients are elderly with numerous comorbidities and conditions that may have an additional role in the pathogenesis of the anaemia including the high risk of infections [4]. Interestingly, it has been also recently demonstrated that CD71+ erythroid cells suppress T cell function contributing to impaired immune function observing that anaemia with an increasing of immature CD71+ erythroid cells are associated multiple myeloma progression in a preclinical MM model [5^▪^].

However, an emerging aspect in the pathophysiology of anaemia in MM patients is the treatment related effect of the new drugs as immunomodulatory drugs (IMiDs), anti-CD38 mAbs and the other immunotherapeutic drugs as CAR-T cells and bispecific antibodies [6] that are recently introduced in the therapeutic armamentarium of MM. 

**Box 1:**
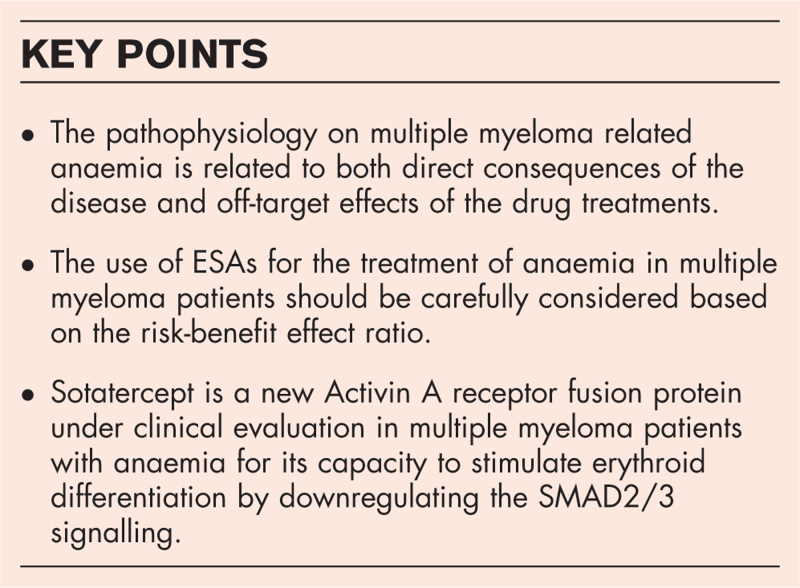
no caption available

## IMMUNOTHERAPY-INDUCED ANAEMIA

### Immunomodulatory drugs

Lenalidomide and pomalidomide, are cornerstone therapies in multiple myeloma treatment, significantly improving progression-free survival (PFS) and overall survival (OS) across newly diagnosed and relapsed settings. Despite their clinical efficacy, immunomodulatory drugs (IMiDs) are associated with significant haematological toxicity, (particularly anaemia, which can compromise dose intensity), treatment interruptions, and low quality of life [7]. More recently, next-generation cereblon E3 ligase modulators (CELMoDs), such as iberdomide, have demonstrated potent antimyeloma activity, even in patients who are heavily pretreated or refractory to IMiDs. Despite their efficacy, CELMoDs continue to carry a risk of bone marrow suppression.

**FIGURE 1:**
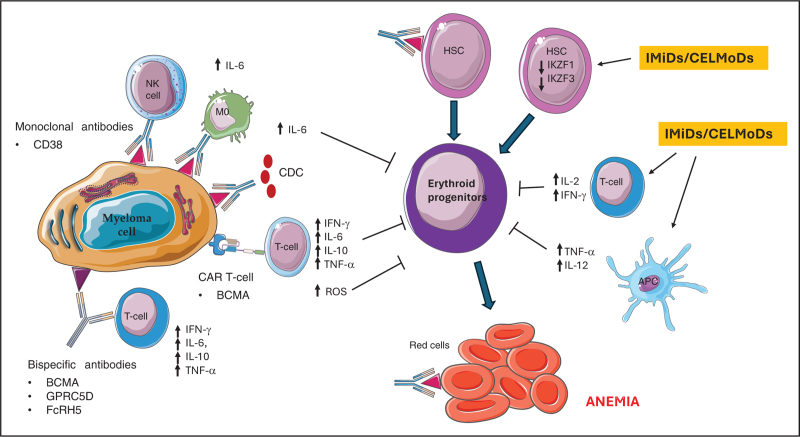
Mechanisms involved in anemia induced by treatment in multiple myeloma pateints. Anaemia associated with IMiDs results from both direct effect on hematopoietic progenitor cells and indirect one mediated by cytokine modulation within the bone marrow microenvironment. Evidence indicates that anaemia induced by anti-CD38 monoclonal antibodies may result from multiple mechanisms, including direct BM suppression and possible haemolysis due to the presence of the CD38 antigen on red cells. Secondly, anti-CD38 mAbs deplete CD38+ immune regulatory cells, including subsets of T and B lymphocytes that may indirectly influence erythropoiesis by altering cytokine profiles. CAR T-cells and bispecific antibodies, release a high number of pro-inflammatory cytokines, that are involved in the suppression of the function of erythroid progenitor cell populations. Moreover, the accumulation of the reactive oxygen species iproduced by CAR T cells can also lead to impaired haematopoiesis. BCMA, B-cell maturation antigen; CDC, complement-dependent cytotoxicity; CELMoD, cereblon E3 ligase modulatory drugs; FcRH5, Fc receptor-like 5; GPRC5D, G protein–coupled receptor class C group 5 member D; HSC, haemopoietic stem cells; IFN, interferon gamma; IKZF, Ikaros Zinc Finger; IL, interleukin; IMiDs, immunomodulatory drugs; M0, monocytes; NK, natural killer; ROS, reactive oxygen species; TNF, tumour necrosis factor.

Anaemia associated with IMiDs and CELMoD therapy results from both direct effects on hematopoietic progenitor cells and indirect effects mediated by cytokine modulation. increasing the production of IL-2 and interferon (IFN)-γ within the bone marrow microenvironment [7,8] that can be involved in the pathogenies of anaemia in MM patients treated with these drugs. The pro-inflammatory IFN-γ production drives acute anaemia by promoting red blood cell (RBC) clearance by activated macrophages IFN-γ also disturbs iron homeostasis and thereby the erythroid balance by inducing iron retention in macrophages. Furthermore, erythroid colony formation of haemato-poietic progenitors is also directly suppressed by IFN-γ that inhibits the earliest stages of erythroid differentiation and proliferation [9] (Fig. 1).

The myeloid transcription factors IKZF1 and IKZF3 play critical roles in normal haematopoiesis; their degradation induced by IMiDs can impair erythroid and myeloid lineage differentiation, contributing to cytopenias. Lenalidomide induces degradation of IKZF1 and IKZF3, affecting lymphoid and erythroid precursors. Additionally, bone marrow suppression may be exacerbated in pts with prior or concurrent therapy such as proteasome inhibitors or anti-CD38 antibodies.

Clinically significant anaemia occurs, especially in heavily pretreated or renally impaired pts. Studies show that lenalidomide can provoke grade 3–4 anaemia in up to 15–20% of treated pts but this frequency is lower in maintenance therapy [7,8,10]. Rare cases of severe aplastic anaemia have also been described [11].

Pomalidomide demonstrates greater potency than lenalidomide [12]. In the MM-003 trial, which evaluated pomalidomide in relapsed/refractory (RR) MM patients previously exposed to lenalidomide and bortezomib, grade at least 3 anaemia occurred in approximately 22–28% of patients [12]. Real-world analyses have confirmed these findings, with some studies reporting anaemia rates as high as 30–40% in heavily pretreated populations [13]. Alternate-day dosing has been demonstrated to mitigate hematologic toxicity; this approach appears to reduce severe cytopenias while preserving antimyeloma activity [14,15]. Because anaemia risk varies according to baseline bone marrow reserve, treatment history, and coexisting medical conditions, dosing strategies should be tailored to the individual patient.

Iberdomide (CC-220) exhibits significantly increased cereblon affinity, resulting in more profound degradation of IKZF1 and IKZF3, and demonstrates potent antimyeloma activity even in pts refractory to prior IMiD therapy [16,17]. In the CC-220-MM-001 trial involving heavily pretreated pts, grade at least 3 anaemia occurred in approximately 28% of patients [18]. Similar rates of severe anaemia have been observed in combination regimens, such as iberdomide–daratumumab–dexamethasone. By contrast, lower iberdomide doses employed in maintenance therapy after autologous stem-cell transplantation, as studied in the EMN26 trial, were associated with minimal grade at least 3 anaemia (0–5%) [19]. Despite the efficacy, iberdomide can still cause marrow suppression, emphasizing the importance of balancing therapeutic potency with hematologic tolerability, especially in heavily pretreated pts or those receiving combination regimens [20].

Management strategies for drug-induced anaemia include dose interruptions or reductions, alternate-day dosing, growth factor support, and careful monitoring of haematological parameters [14,15]. Tailoring therapy based on patient risk factors, prior treatment history, and response is essential to optimize both efficacy and safety, particularly in older or heavily pretreated pts. Emerging data suggest that using lower CELMoD doses in maintenance or in combination regimens may maintain antimyeloma activity while minimizing haematologic toxicity [15,16].

In summary, IMiDs and CELMoDs form the backbone of MM therapy, with potent antimyeloma activity mediated via cereblon-dependent mechanisms. However, their clinical utility is frequently limited by haematological toxicities, particularly anaemia [12]. Severity increases from lenalidomide to pomalidomide, to iberdomide in high-dose RRMM regimens. Understanding mechanisms, clinical incidence, and management of these toxicities is critical for maximizing therapeutic benefit while preserving quality of life [12,14–16]. Future research should continue to refine dosing schedules, explore predictive biomarkers for toxicity, and optimize combination regimens to balance potency with haematologic tolerability.

### Anti-CD38 monoclonal antibodies 

Anti-CD38 monoclonal antibodies (mAbs) such as Daratumumab and Isatuximab have significantly improved outcomes in MM patients. Anti-CD38 mAbs are generally effective and well tolerated. At the same time, haematological adverse events – particularly anaemia – remain clinically relevant [21,22]. Anaemia in patients treated with anti-CD38 mAbs, needs to be understood in its pathophysiology, prevalence, and management [23].

The exact pathophysiology of anaemia in MM patients treated with anti-CD38 mAbs remains incompletely understood. Anaemia in MM patients is likely multifactorial and multiple mechanisms have been proposed. These mechanisms involve both direct drug effects and indirect mechanisms like patient-specific factors. Understanding the mechanisms of anaemia is critical for optimizing therapy.

The main direct mechanism of anaemia is because CD38 is expressed not only on plasma cells but also on haematopoietic progenitors, particularly erythroid precursors. This suggests that anti-CD38 antibodies may induce apoptosis or functional inhibition of these progenitors, leading to transient reductions in erythropoiesis [24–26]. Indeed, clinical evidence from transplant studies shows delayed red cell engraftment in patients pretreated with daratumumab, thus supporting a direct inhibitory effect on erythroid precursors [24,25].

Secondly, anti-CD38 mAbs deplete CD38+ immune regulatory cells, including subsets of T and B lymphocytes. This modulation of the immune microenvironment may indirectly influence erythropoiesis by altering cytokine profiles (especially IL-6) [27] and reducing erythropoietin responsiveness [28]. Complement activation and Fc-mediated cytotoxicity may induce a pro-inflammatory milieu that transiently suppresses bone marrow erythropoietic activity [28].

In addition, concomitant therapies, including iIMiDsor proteasome inhibitors, may exacerbate anaemia via myelosuppressive effects. Alkharabsheh *et al.* [23] reported grade 3–4 anaemia in RRMM patients treated with daratumumab plus pomalidomide and dexamethasone (DPd) in approximately 18% of cases. Also, in the ICARIA-MM phase III trial, grade at least 3 anaemia occurred in 25–30% of pts receiving Isatuximab-Pomalidomide and dexamethasone (IsaPd), largely attributable to pomalidomide's myelosuppressive effects [29]. While in the IKEMA study, grade at least 3 anaemia occurred in 16–24% of patients receiving Isatuximab-Carlfizomib and dexamethasone (IsaKd) [30] and Carfilzomib is recognized as a contributor to anaemia.

Moreover, recent data indicate a high prevalence of iron deficiency in newly diagnosed MM patients treated with daratumumab. The iron deficiency is possibly due to inflammatory hepcidin-mediated iron sequestration or subclinical blood loss. This could suggest that iron metabolism alterations may contribute to anaemia independent of disease burden [28]. The recognition of iron deficiency as a contributing factor is particularly important, as this is potentially correctable with supplementation. Clinicians should monitor haemoglobin, ferritin and transferrin saturation, especially in patients receiving daratumumab-based regimens.

Furthermore, it is important to remember that anti-CD38 mAbs can interfere with indirect antiglobulin tests, leading to transfusion delays and functional anaemia [31]. Indeed, Daratumumab binds to CD38 molecules that are weakly expressed on reagent RBCs used in pretransfusion testing. This leads to pan-reactivity in antibody screening and compatibility assays, thereby mimicking the presence of alloantibodies or autoantibodies [32]. This serological interference does not represent true immune-mediated haemolysis. Several studies have shown that despite persistent Coombs test positivity, pts receiving daratumumab rarely exhibit biochemical or haematological markers of haemolysis [33]. Consequently, a positive Coombs test in this clinical context must be interpreted with caution and always correlated with clinical findings and laboratory evidence of haemolysis.

In contrast, few anecdotal case reports suggest daratumumab activity in other types of anaemia such as autoimmune haemolytic anaemia (AIHA) and pure red cell aplasia (PRCA). Refractory immune-mediated anaemia is thought to result from the persistence of plasma cells that continue producing isohemagglutinins. By targeting and eliminating these pathogenic plasma cells, daratumumab may be responsible for its remarkable efficacy in such conditions [32].

The optimal management of anti-CD38 mAbs-associated anaemia requires a multifaceted approach. In fact, it is important to monitor haemoglobin assessment and iron studies are recommended. The iron supplementation is indicated in documented deficiency, while transfusion support remains the standard therapy for symptomatic anaemia [31]. Dose modification of concomitant myelosuppressive agents may be required for persistent or severe anaemia. Consequently, prospective studies are needed to quantify the direct effect of anti-CD38 mAbs on erythroid progenitors and to find a biomarker-driven approach. A personalized management with alternative dosing strategies, including low-intensity or extended-interval regimens, may reduce haematologic toxicity while maintaining efficacy [33].

### Bispecific antibodies and CAR T cell therapies

T-cell redirecting bispecific antibodies (BsAbs) and chimeric antigen receptor T cells (CAR T-cells) have significantly modified the natural history of multiple myeloma patients [34]; their use is constantly increasing in rRRMM patients, and several trials are ongoing also in newly diagnosed MM.

BsAbs and CAR T-cells treatments, in multiple myeloma as well as in all haematological malignancies, are characterized with a specific and unique profile of toxicities; although the most well known and studied are cytokine release syndrome (CRS) and immune effector cell-associated neurotoxicity syndrome (ICANS), haematological toxicities are so far the most commons grade at least 3 adverse events [35^▪▪^].

For BsAbs (Teclistamab, Talquetamab and Elranatamab) the incidence of anaemia reported in a European myeloma Network (EMN) consensus ranges from 45 to 67%, with an incidence of grade 3 or higher anaemia up to 50%. A recent pooled analysis on 2374 multiple myeloma patients treated with BsAbs showed the occurrence of anaemia in 39.2%, with 24.5% grade at least 3 anaemia. Interestingly, the analysis showed higher incidence of anaemia in patients treated with B-cell maturation antigen (BCMA)-directed BsAbs compared to those treated with GPRC5D/FcRH5 BsAbs [36].

Regarding CAR T-cells, Idecabtagene vicleucel (ide-cel), a BCMA CAR T-cell therapy, has been approved by American and European regulatory agencies on the basis of the results of the KARMMA-3 trial; the trial reported an incidence of all-grade anaemia of 66% and of grade 3–4 anaemia of 51% [37].

Ciltacabtagene vicleucel (cilta-cel), another BCMA CAR T cell therapy, has also been approved by the Food and Drug Administration (FDA) in the United States and by the European Medicine agency (EMA) based on the results of the phase III CARTITUDE-4 study. The trial reported and incidence of all-grade anaemia of 54.3% (grade 3–4 35.6%) [38].

Recently, the European Hematology Association (EHA) and the European Society for Blood and Marrow Transplantation (EBMT) have been renamed cytopenias subsequent to CAR T cell therapy as immune effector cell-associated haematological toxicity (ICAHT) [39]. Cytopenias subsequent to CAR T-cell can be classified into three distinct groups based on their onset and duration into early cytopenias (0–30 days), long-term cytopenias (30–90 days) and late cytopenias (after 90 days).

The exact mechanism that leads to the onset of cytopenias, and particularly anaemia during immunotherapies is not completely understood. Regarding CAR T-cell, several risk factors has been associated with the onset of cytopenias: baseline characteristic, such as number of prior lines of therapy, elevated disease burden, or a history of previous allogeneic stem cell transplantation [40]. Conditioning chemotherapy prior to CAR T-cell reinfusion can also led to the occurrence of early cytopenias [41].

To identify patients at higher risk of developing prolonged cytopenias, a score initially applied to patients with lymphoma has been validated also in patients with MM [42]. The CAR-HEMATOTOX score is calculated before lymphodepletion and is based on factors related to the hematopoietic reserve (haemoglobin level, platelets count, absolute neutrophil count) and factors related to the baseline inflammatory state (C-reactive protein, ferritin) [43]. In the study, the score has demonstrated a high potential of identifying MM patients treated with CAR T-cell at higher risk of developing cytopenias; patients with the higher score, moreover, have significantly higher incidence of nonrelapse mortality, predominantly due to fatal infections, and shorter progression-free survival (PFS).

Inflammatory status has also been associated to the onset of cytopenia, and there are several evidence that relates the severity of cytopenias with the onset of CRS. CAR T-cells, after engagement with tumour cells, release a high number of pro-inflammatory cytokines, such as IFN-γ, IL-6, IL-10 and TNF-α. Of those, IL-6 reduces the function of erythroid progenitor cell populations [40,44]. High serum concentrations of IL-6, IFN-γ and IL-10 have been identified as risk factors for the occurrence of cytopenias [45]. Other mechanisms that are hypothesized to be involved in the development of cytopenia include the accumulation of reactive oxygen species (ROS) in the bone marrow produced by CAR T cells, that can lead to impaired haematopoiesis [46] and the presence of clonal haematopoiesis in the bone marrow. It has been observed, indeed, that up to 48% of patients eligible for CAR T therapy had a CHIP clone in the bone marrow [47].

The mechanism leading to the onset of anaemia during treatment with BsAbs is not well understood, but factors like those involved in CAR T-cell therapy appear to play a role.

## TREATMENT OF ANAEMIA IN MULTIPLE MYELOMA PATIENTS

Because the anaemia is mainly related to the disease activity, MM targeted treatment is the first therapeutic option [48,49]. However supportive care is also included based on the use of RBC transfusions and erythropoietin-stimulating agents (ESAs). A restrictive transfusion cut offs is considered a level of haemoglobin of 7 g/dl as supported by a Cochrane review [50].

ESAs, such as epoetin or darbepoetin alpha, increase haemoglobin levels [51]. However, ESAs may increase the risk of thromboembolic events in MM patients, especially in combination with IMiDs [51–53]. Combined ASCO and ASH practice guideline have suggested that ESAs may be offered to cancer patients with chemotherapy-associated anaemia to reduce the need for RBC transfusions, but when cancer treatment is not curative [54].

Therefore, treatment with ESAs should follow international guideline recommendations with a particular attention in elderly frail patients for the risk of thrombosis [55]. The goal of the of the ESAs is to reduce the need of RBC transfusions. Interestingly, an Italian small clinical study in elderly patients with MM evaluated the effect of biosimilar epoietin alpha showing an improvement of post treatment haemoglobin with a significant impact on the quality of life [56].

Because the high incidence of anaemia during the new immunotherapies as CAR-T cells and bispecific, it is essential for clinicians to be careful in monitoring and managing this adverse event to prevent major complications and worsening quality of life for patients. All patients with anaemia should receive supportive transfusions when symptomatic or with Hb values less than 7 g/dl. ESAs can be considered in patients with persistent anaemia to haemoglobin less than 10 g/dl [57]. For prolonged and late cytopenias, stem cell boost can also be considered [58].

### Activin receptor fusion proteins

In the recent years, alternative strategies against multiple myeloma associated anaemia by targeting the activin signalling pathway. The role of TGF-beta/SMAD signalling pathway in hematopoietic stem cell development and homeostasis is well known [59]. TGF-beta receptors can be categorized in two different types, and several ligands have been identified including activins, inhibin, bone morphogenetic proteins (BMPs) and growth and differentiation factors (GDFs) [60]. Activin-A can activate receptors formed by ActRI and ActRII [60]. Activin receptor type IIa ligand traps (fusion proteins), such as sotatercept and luspatercept are recognized to enhance erythroid differentiation by downregulating the SMAD2/3 signalling in erythroid progenitor cells [61] and they are approved in the treatment of myelodysplastic syndrome. Sotatercept have high affinity for different TGF-beta ligands such as Actinin A, Activin B, GDF11 and BMP-10 [60] and targets BFU-E and late-stage erythroid progenitors promoting erythropoiesis [60,62,63].

In MM patients, the effect of sotatercept has been recently investigated (ACE-011), in combination with melphalan, prednisone and thalidomide (MPT) showing a significant bone anabolic and erythropoietic effect [64]. In the clinical trial, MM patients received subcutaneous injections of 0.1, 0.3, 0.5 mg/kg sotatercept or placebo every 28 days for a total of four cycles, in combination with MPT. The results suggest that sotatercept at medium and high doses can effectively improve Hb levels in multiple myeloma patients, with a dose--response effect. Currently, clinical studies of sotatercept in combination with lenalidomide/pomalidomide and dexamethasone for the treatment of multiple myeloma patients are ongoing (NCT01562405) waiting for the results.

Finally, a novel ALK-2 inhibitor, INCB000928, is currently assessed in a phase 1/2 trial in patients with myelodysplastic syndrome or MM who are transfusion-dependent or have symptomatic anaemia (NCT04582539).

## CONCLUSION

Anaemia is a common feature in MM patients due to multifactorial mechanisms. Apart from the well known factors related to the disease, more recently the use of several new drugs as the immunotherapeutic ones indicates the emerging role of drug related mechanisms in the pathophysiology of anaemia in MM patients. Anaemia associated with IMiDs results from both direct effect on hematopoietic progenitor cells and indirect one mediated by cytokine modulation within the bone marrow microenvironment. Anti-CD38 monoclonal antibodies are an effective therapy for multiple myeloma, but anaemia remains a clinically significant adverse event. Evidence indicates that anaemia may result from multiple mechanisms, including bone marrow suppression, iron deficiency and concomitant therapy effects. In addition, recent clinical trials reported ad high incidence of anaemia in multiple myeloma patients treated either with bispecific antibodies or CAR-T cells mainly due to a pro-inflammatory status into the bone marrow microenvironment. CAR T-cells, release a high number of pro-inflammatory cytokines, such as IFN-γ, multiple myeloma -6, IL-10 and TNF-that are involved in the suppression of the function of erythroid progenitor cell populations [44].

Figure 1 summarizes the main mechanisms involved in drug-induced anaemia in MM patients.

Drug-related anaemia should be considered by clinicians particularly in the management of those patients in which coexist other anaemia-related factors as those with renal insufficiency and in frail elderly patients.

Treatment of anaemia in multiple myeloma patients included the used of RBC transfusion and ESAs but recently novel agents were under investigation as the activin receptor fusion proteins as sotatercept. Further clinical studies are necessary to demonstrate clinical benefit of these new agents

## Acknowledgements

*The author conveys thanks to Paola Storti and Nicolas Thomas Iannozzi for the technical support.*

*Figure 1 was generated from adapted figures provided by Servier Medical Art (Servier;*https://smart.servier.com/), licensed under a Creative Commons Attribution 4.0 Unported License.

### Financial support and sponsorship

*None.*

### Conflicts of interest

*There are no conflicts of interest.*

## REFERENCES AND RECOMMENDED READING

Papers of particular interest, published within the annual period of review, have been highlighted as:

▪ of special interest
▪▪ of outstanding interest
